# Neighborhood environment and muscle mass and function among rural older adults: a 3-year longitudinal study

**DOI:** 10.1186/s12942-020-00247-9

**Published:** 2020-11-25

**Authors:** Kenta Okuyama, Takafumi Abe, Shozo Yano, Kristina Sundquist, Toru Nabika

**Affiliations:** 1 Center for Primary Health Care Research, Lund University, Jan Waldenströms gata 35, 20502 Malmö, Sweden; 2 Center for Community-Based Healthcare Research and Education (CoHRE), Organization for Research and Academic Information, Shimane University, 223-8 Enya-cho, Izumo-shi, Shimane, 693-8501 Japan; 3 Department of Laboratory Medicine, Faculty of Medicine, Shimane University, 89-1 Enya-cho, Izumo-shi, Shimane, 693-8501 Japan; 4 Department of Family Medicine and Community Health, Department of Population Health Science and Policy, Icahn School of Medicine At Mount Sinai, 1 Gustave L. Levy Place, New York, NY 10029-5674 USA; 5 Department of Functional Pathology, Faculty of Medicine, Shimane University, 89-1 Enya-cho, Izumo-shi, Shimane, 693-8501 Japan

**Keywords:** Neighborhood environment, Sarcopenia, SMI, Grip strength, Rural

## Abstract

**Background:**

Sarcopenia, resulting from loss of muscle mass and function, is highly prevalent in the ageing societies and is associated with risk of falls, frailty, loss of independence, and mortality. It is important to identify environmental risk factors, so that evidence-based interventions to prevent sarcopenia can be implemented at the population level. This study aimed to examine the potential effect of several objectively measured neighborhood environmental factors on longitudinal change of muscle mass and function among older adults living in rural Japanese towns where the population is ageing.

**Methods:**

This study was based on data from the Shimane CoHRE Study conducted by the Center for Community-based Healthcare Research and Education (CoHRE) at Shimane University in 3 rural towns in the Shimane Prefecture, Japan. Subjects older than 60 years, who participated in an annual health examination in 2016 and any follow-up years until 2019, i.e., 4 possible time points in total, were included (n = 2526). The skeletal muscle mass index (SMI) and grip strength were assessed objectively for each year as a measure of muscle mass and function, respectively. Neighborhood environmental factors, i.e., hilliness, bus stop density, intersection density, residential density, and distance to a community center were measured by geographic information systems (GIS). Linear mixed models were applied to examine the potential effect of each neighborhood environmental factor on the change of SMI and grip strength over time.

**Results:**

Males living far from community centers had a less pronounced decline in SMI compared to those living close to community centers. Females living in areas with higher residential density had a less pronounced decline in grip strength compared to those living in areas with lower residential density.

**Conclusions:**

Neighborhood environmental factors had limited effects on change of SMI and grip strength among rural older adults within the 3 years follow up. Further long-term follow up studies are necessary by also taking into account other modifiable neighborhood environmental factors.

## Introduction

Sarcopenia is defined as a progressive and generalized skeletal muscle disorder resulting from loss of muscle mass and muscle function and is associated with risk of falls, frailty, loss of independence, and mortality among older adults [1–4]. Several factors, such as physical activity, diet, and smoking have been identified as potential risk factors for sarcopenia [5–8]. While these individual modifiable factors are important in interventions at the individual level, neighborhood-level factors may also be key in the prevention of sarcopenia at the population level because of their potential to determine people’s behaviors and subsequent health outcomes. Neighborhood environments may be of importance particularly for older adults, who are likely to spend more time in their neighborhoods of residence than younger persons [9]. Several studies have examined the association between neighborhood environmental factors, e.g., safety, physical disorder (trash or littering), and social cohesion and older adults’ physical functions such as activities of daily living [10–14]. One study from the US found that living in neighborhoods with high physical disorder, low social cohesion and low safety was associated with higher incidence of limitations in activities of daily living, such as getting across the room, bathing, eating and shopping for groceries [14]. Other studies from the UK and Australia have found that living in neighborhoods with low socioeconomic status is associated with poorer physical function measured by self-reported functional limitations in different daily activities [10–12, 15]. One study from the US assessed physical function objectively and found that self-reported neighborhood disorder was associated with lower physical function [13]. However, no previous studies have assessed both neighborhood environments and physical functions based on objective measures, such as functions related to sarcopenia. Sarcopenia has been defined as a clinical disorder in WHO’s International Classification of Diseases (ICD) version 10 (ICD-10-CM (M62.84)) in 2016 and is based on objectively measured muscle mass and function [16]. There is firm evidence that these objective measures are associated with falls, frailty, loss of independence, and mortality [17–22]. Given this, studies should also strive to identify neighborhood environmental factors that may affect objectively measured muscle mass and function, in order to find avenues for prevention and environmental interventions to avoid sarcopenia at the population level. Sarcopenia is currently estimated to amount to a prevalence of 10–40% and is therefore associated with high healthcare costs [23, 24]. Current findings for neighborhood environmental factors associated with physical function are also limited to urban western settings with a cross-sectional design [25]. Furthermore, while its association might differ by gender, few studies have analyzed males and females separately and with a sufficient sample size.

Considering this background, our study aimed to investigate whether objectively measured neighborhood environmental factors may affect objectively measured muscle mass and function among older adults by using a longitudinal study design within rural towns of Japan where the population is ageing.

## Methods

### Study design

This 3-year longitudinal study, using data from the Shimane CoHRE Study, was conducted by Shimane University in collaboration with 3 municipalities in Shimane Prefecture. The Shimane CoHRE Study aims to prevent lifestyle-related diseases by collecting data on medical information, lifestyle, physical function, and neighborhood environment during annual municipal health examinations. The present study used data between 2016, when the study began and 2019 to measure muscle mass and function. Informed consent was obtained from all subjects before enrolment. This study was conducted with the approval of the Ethics Committee of Shimane University School of Medicine (#20180420-2).

### Study subjects

**Fig. 1:**
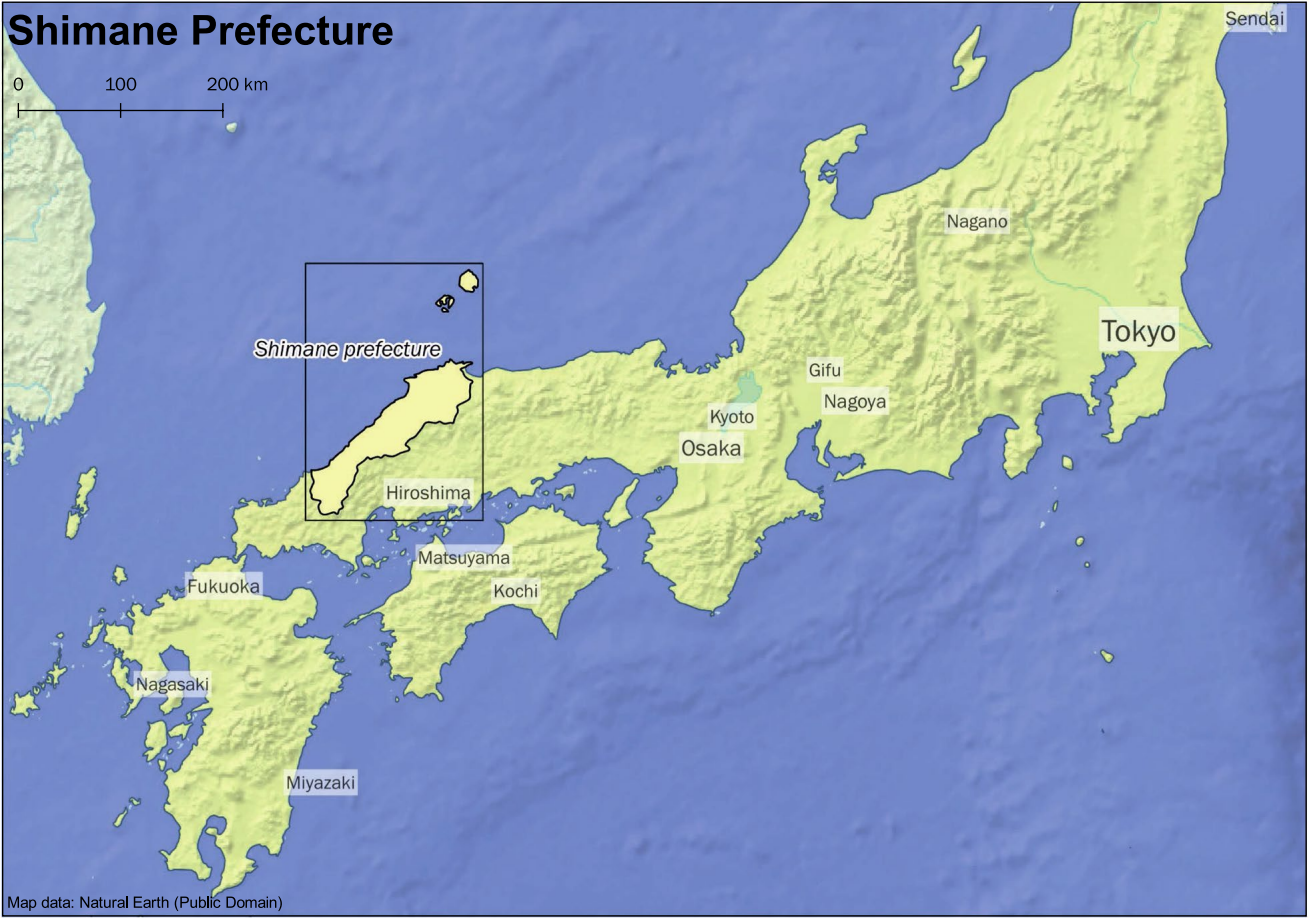
Study area: Shimane Prefecture in Japan

**Fig. 2:**
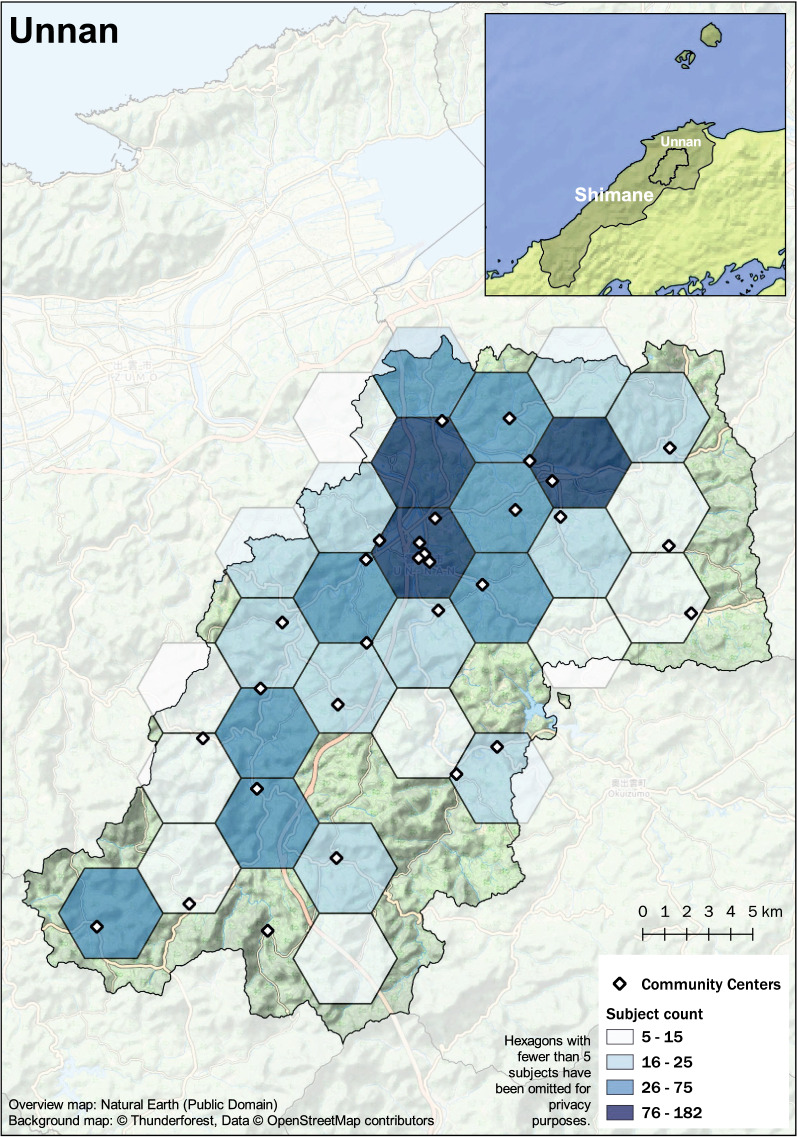
Distribution of study samples in Unnan, Shimane

**Fig. 3:**
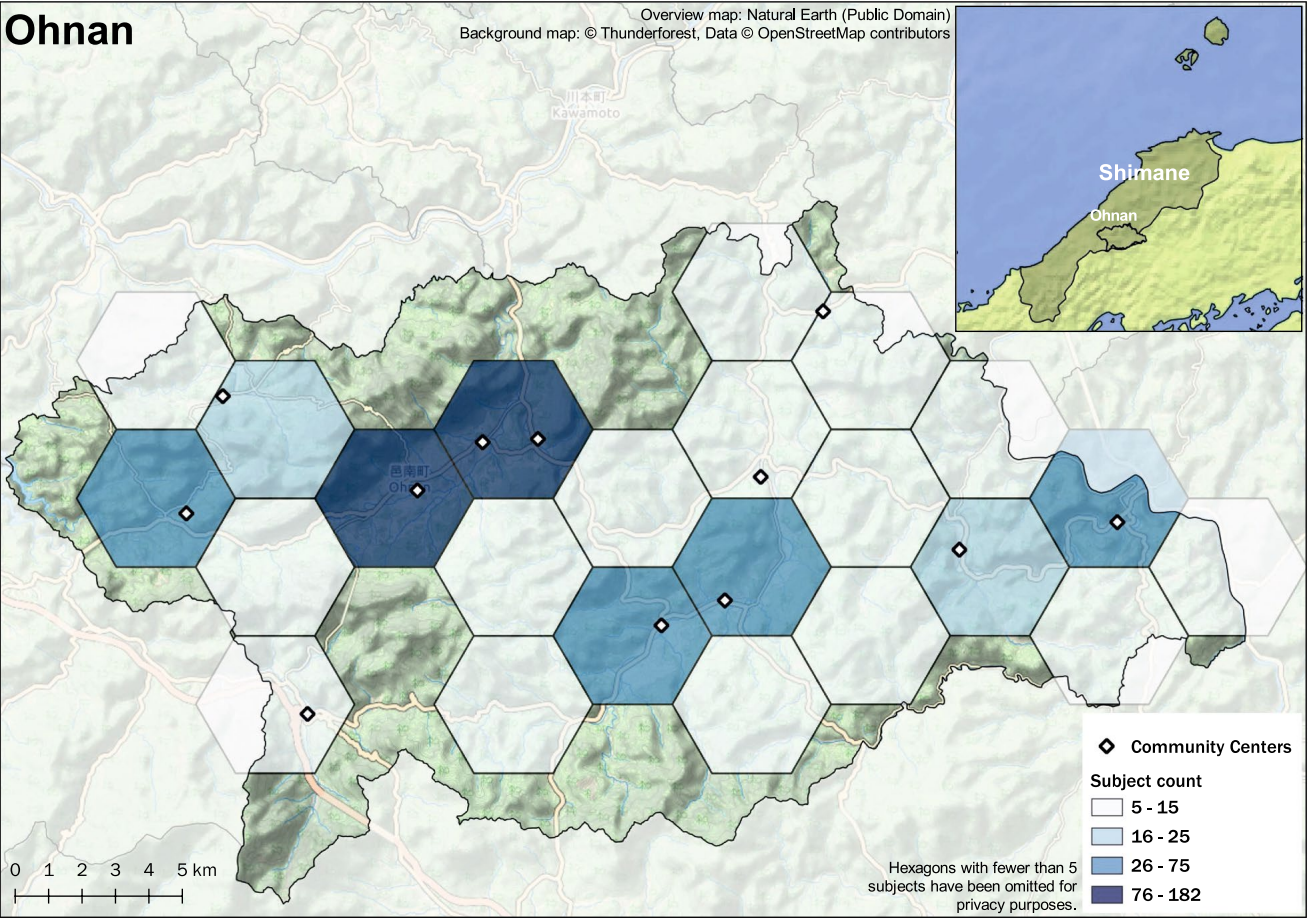
Distribution of study samples in Ohnan, Shimane

**Fig. 4:**
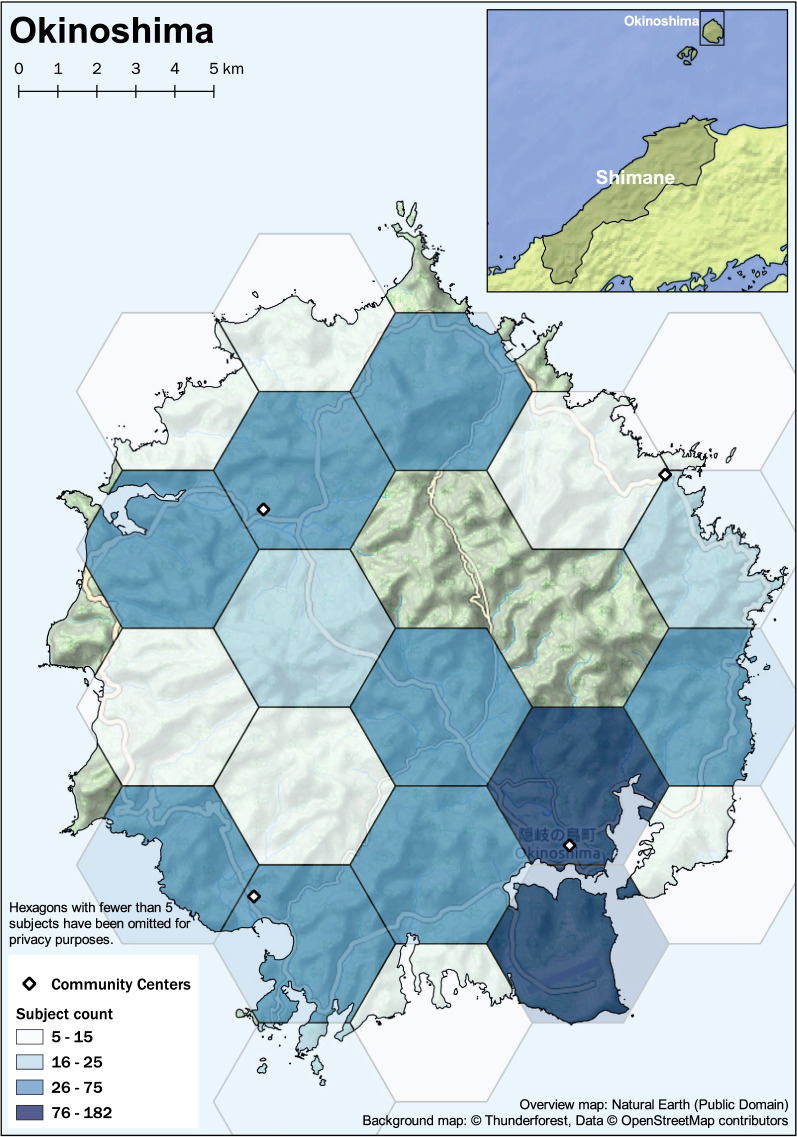
Distribution of study samples in Okinoshima, Shimane

Rural mountainous regions of Japan, which feature a unique environment, with a proportion of an older adult population above approximately 35% were subject in this study. Subjects were older adults over the age of 60 years residing in the towns of Ohnan, Okinoshima, and Unnan in the Shimane Prefecture as shown in the maps in Figs. 1, 2, 3, and 4. Eligibility for this study was participation in the 2016 baseline survey, which measured muscle mass and function and participation in any of the follow-up surveys during the 3 years between 2017 and 2019. The final analysis included 2526 subjects (1013 males and 1513 females). Exclusion was performed based on the following criteria: 43 subjects with missing skeletal muscle mass index (SMI) values at baseline, 13 subjects with missing grip strength values at baseline, and 30 subjects for whom mean land slope angles could not be calculated due to the resolution of the data.

### Outcome variable

Limb muscle mass was measured with a body composition analyzer using bioimpedance method (MC-780A, Tanita Corporation, Tokyo, Japan). Skeletal muscle mass index (SMI) was calculated based on limb skeletal muscle mass divided by the square of body height. Muscle function was assessed based on the maximum grip strength measured by two attempts in each hand. Higher levels of muscle mass and muscle function are related to better health [1–4]. We examined continuous changes in SMI and maximum grip strength over time. As a sensitivity analysis, we examined the number of new sarcopenia cases between 2016 and 2019. According to the gender-specific Asian sarcopenia criteria, we classified those having sarcopenia as follows: SMI in males < 7.0 kg/m^2^ and females < 5.6 kg/m^2^ and maximum grip strength in males < 28 kg and females < 18 kg [26]. For this analysis, we created a different subset of the cohort by excluding subjects who had sarcopenia already in the first year of participation (2016, 2017, or 2018), and included those who had more than 2 measurements between 2016 and 2019.

### Exposure variables

Hilliness, bus stop density, intersection density, residential density, and distance to community centers were measured by geographic information systems (GIS). The measures for hilliness, bus stop density, intersection density and residential density were calculated within a 1000 m network buffer from the point of residence of each subject based on actual street network. We used 1000 m network buffers, which has been found to be an appropriate space for activities in previous neighborhood studies [27]. Land slope was used to assess the hilliness of the neighborhood. Hilly neighborhoods have been reported to be associated with both higher and lower physical activity and weight gain in older adults [28–30]. The mean land slope was computed based on the Elevation and degree of Slope 5th Mesh Data obtained from the National Land Numerical Information (NLNI), which is publicly available GIS data administered by the National Land Information Division, National Spatial Planning, and Regional Policy Bureau of Japan. We calculated the mean land slope for each individuals’ network buffer by the value of the slope stored in each 50 m grid (5th Mesh Data) which intersected with network buffers. Higher bus stop density, intersection density and residential density have been reported to be associated with higher physical activity levels and better health in older adults [31, 32]. Both bus stop and intersection density were calculated based on the number of points, i.e., bus stops and intersections with three or more legs within network buffers. Point data of bus stops was obtained from the Detailed Map, ArcGIS data collection, and point data of intersections was obtained from the Street Network, ArcGIS data collection administered by Esri Corporation, Tokyo, Japan (Esri Japan). Residential density was calculated using the number of households located in the smallest Japanese census statistics areas within network buffers, which was also obtained as point data from the ArcGIS data collection from Esri Japan. Distance to a community center was calculated based on street networks from each point of residence to the community center of the subject’s community district. Community centers in Japan are public facilities that provide educational, cultural and other recreational activities to the community. It is also a place for local associations, such as senior citizens’ clubs, to organize regular meetings. Previous research has shown that participating in social activities at community centers contributes to the maintenance of activities of daily living among older adults [33]. The calculated values were categorized by using medians into two groups (high, low or close, far), and their effects on the changes in SMI and maximum grip strength over time were analyzed. All spatial analyses were done by ArcGIS Pro 2.0 (Esri Japan).

### Covariates

Factors that could potentially confound or modify the association between neighborhood environmental factors and SMI and grip strength, including age, residential town, smoking, drinking, physical activity habits, musculoskeletal disorders, cardiovascular disease, and cerebrovascular disease, were obtained through self-administered questionnaires and were included in the analysis. Lifestyle factors such as smoking, drinking and physical activity are associated with neighborhood environmental factors [31, 32], as well as risk factors of sarcopenia [3]. Chronic conditions such as musculoskeletal disorders, cardiovascular disease and cerebrovascular disease are known risk factors of sarcopenia [3]. Therefore, they were included as adjustment variables in the analyses of the association between neighborhood environmental factors and SMI and grip strength.

### Statistical analysis

Frequency and proportion for the subjects’ baseline characteristics are described by gender. Changes in SMI and maximum grip strength over time are reported as the mean and standard deviation by gender, age, and residential town. The influence of neighborhood environmental factors on SMI and maximum grip strength was analyzed using a linear mixed effects model. The linear mixed effects model estimates the intra-individual variation as random effects when repeated measures of individual data are available to estimate the fixed effect of the explanatory variable on the target variable over time [34]. Whether to include the intercept and slope of the individual as random effects was determined by the Akaike Information Criterion (AIC). We used a model that estimated both the intercept and the slope as a random effect for all analyses. All neighborhood environmental factors were included separately in the models to avoid multi-collinearity (correlation analysis was conducted and is shown in Additional file 1: Tables S1, S2). Model 1 included only time, i.e., year for assessment of change of outcomes. Model 2 also included the exposure, i.e., the neighborhood environmental factors, to assess the change of outcomes adjusting for all the covariates. As a sensitivity analysis, we described the number of new sarcopenia cases between 2016 and 2019. We used R 3.6.1, an open source statistical tool for the analysis, and the threshold for statistical significance was set to < 0.05.

## Results

### Subject characteristics

The final analysis included 2526 subjects (1013 males and 1513 females), characterized by the factors included in the self-administered questionnaire (Table 1).

**Table 1:** Characteristics of study subjects at the baseline (2016) by gender

	Male	Female
n	1013	1513
Age = 60–75/76 + (%)	712/301 (70.3/29.7)	1073/440 (70.9/29.1)
Town of residence (%)		
Unnan	492 (48.6)	625 (41.3)
Oki	261 (25.8)	512 (33.8)
Onan	260 (25.7)	376 (24.9)
Smoking status = Yes (%)	159 (15.7)	13 (0.9)
Drinking status (%)		
Yes	532 (52.5)	133 (8.8)
Occasionally	183 (18.1)	280 (18.5)
No	298 (29.4)	1100 (72.7)
Physical activity habit = Yes (%)	524 (51.7)	760 (50.2)
Musculoskeletal disorders = Yes (%)	153 (15.1)	486 (32.1)
Cardiovascular disease history = Yes (%)	109 (10.8)	114 (7.5)
Cerebrovascular disease history = Yes (%)	65 (6.4)	45 (3.0)

### Temporal changes in SMI and maximum grip strength

In both males and females, simple aggregated means of both SMI and grip strength were higher among younger age groups, i.e., 60–75 years old, compared to older age group, i.e., 76 years and above at all year points. Mean SMI and grip strength were both higher among males. In the respective gender and age groups, simple aggregated mean by year showed that there was no apparent change in SMI or maximum grip strength over time (Table 2).

**Table 2:** Mean SMI and grip strength across 4 waves by age and towns

		SMI, mean (sd)				Grip strength, mean (sd)			
		2016	2017	2018	2019	2016	2017	2018	2019
Male									
Unnan	60–75	7.6 (0.9)	7.7 (0.9)	7.6 (0.8)	7.6 (0.9)	37.2 (5.7)	37.3 (5.8)	37.1 (5.9)	37.1 (5.1)
	76 +	7.4 (0.8)	7.2 (0.7)	7.3 (0.8)	7.2 (0.8)	32.4 (5.8)	32.8 (5.7)	33.5 (4.4)	33.6 (4.8)
Oki	60–75	7.8 (1)	7.8 (1)	7.7 (0.9)	7.8 (0.9)	38.9 (6.2)	37.6 (5.7)	37 (5.9)	38.7 (6.5)
	76 +	7.5 (0.8)	7.4 (0.9)	7.4 (0.9)	7.4 (0.9)	33.8 (5.7)	33.4 (5.2)	32 (5.5)	32.9 (5.7)
Onan	60–75	7.8 (1)	7.9 (1.1)		7.7 (1)	37.2 (5.4)	36.6 (5.5)		36.4 (5.5)
Female									
Unnan	60–75	6.2 (0.7)	6.2 (0.7)	6.2 (0.7)	6.2 (0.7)	23.7 (3.7)	25.3 (3.7)	24.3 (3.9)	24.1 (3.4)
	76 +	6.1 (0.8)	5.9 (0.6)	5.9 (0.6)	5.9 (0.8)	21 (3.8)	22.2 (4.1)	21.9 (3.9)	22 (3.8)
Oki	60–75	6.4 (0.7)	6.3 (0.6)	6.3 (0.7)	6.4 (0.8)	24.4 (3.6)	23.5 (3.5)	23.9 (3.7)	23.9 (3.8)
	76 +	6.2 (0.8)	6.1 (0.8)	6.1 (0.8)	6 (0.6)	21 (3.7)	20.5 (3.6)	21.2 (4.1)	20.9 (3.7)
Onan	60–75	6.4 (0.7)	6.3 (0.7)		6.4 (0.8)	23.8 (3.9)	24.3 (4.2)		23.6 (3.8)

### Association between neighborhood environment factors, SMI, and grip strength

In males, the linear mixed model using only the year (time) showed that both SMI and maximum grip strength decreased significantly each year ($\upbeta$: − 0.05, 95% CI − 0.06, − 0.04 (SMI)$;\upbeta$: − 0.41, 95% CI − 0.49, − 0.32 (maximum grip strength)) (Table 3). The model, including neighborhood environmental factors and potential confounding factors, showed that SMI was significantly lower in areas with a higher density of bus stops ($\upbeta$: − 0.15, 95% CI − 0.27, − 0.03). This was interpreted as the average effect of bus stop density over time, i.e., assessed at 4 time points from 2016 to 2019. In other words, the coefficient − 0.15 is the difference in SMI between the area with high bus stop density and low bus stop density in average based on the 4 time points. The purpose was to examine how the changes (trajectories) differ in 2017, 2018, and 2019. The change in mean SMI over time was only associated with distance to community center ($\upbeta$: 0.04, 95% CI 0.01, 0.07), which indicated that living far from a community center would alleviate a decline in SMI. With respect to grip strength, an average effect was only found for land slope, i.e., grip strength was significantly higher in areas with a higher land slope ($\upbeta$: 0.93, 95% CI 0.18, 1.68). No significant differences in the change of grip strength over time were found for any of the other environmental factors.

**Table 3:** Male—linear mixed model for SMI and grip strength by environment variable

	SMI		Grip strength	
	Model 1^a^	Model 2^b^	Model 1^a^	Model 2^b^
Variables	β (95% CI)	β (95% CI)	β (95% CI)	β (95% CI)
Year	− 0.05^*^ (− 0.06, − 0.04)	−	− 0.41^*^ (− 0.49, − 0.32)	−
Land slope (ref = Low)		0.01 (− 0.11, 0.14)		0.93^*^ (0.18, 1.68)
Year*Land slope (ref = Low)		0 (− 0.03, 0.03)		− 0.01 (− 0.2, 0.18)
Bus stop density (ref = Low)		− 0.15^*^ (− 0.27, − 0.03)		− 0.33 (− 1.09, 0.43)
Year*Bus stop density (ref = Low)		0.01 (− 0.02, 0.04)		− 0.07 (− 0.26, 0.12)
Intersection density (ref = Low)		0.01 (− 0.11, 0.14)		− 0.49 (− 1.24, 0.26)
Year*Intersection density (ref = Low)		0 (− 0.03, 0.03)		0.13 (− 0.06, 0.31)
Residential density (ref = Low)		− 0.05 (− 0.17, 0.07)		− 0.13 (− 0.89, 0.62)
Year*Residential density (ref = Low)		− 0.01 (− 0.04, 0.02)		0.03 (− 0.16, 0.21)
Distance to community center (ref = Close)		− 0.03 (− 0.15, 0.1)		0.37 (− 0.39, 1.13)
Year*Distance to community center (ref = Close)		0.04^*^ (0.01, 0.07)		− 0.02 (− 0.21, 0.17)

Environment factors were added separately into all models

^*^p < 0.05

^a^Model 1: time only model

^b^Model 2: conditional growth model by each environment factor adjusting for all covariates, i.e. age, town of residence, smoke, drink, physical activity, musculoskeletal disorders, cardiovascular disease, cerebrovascular diseases

In females, like males, the model including the year only showed that both SMI and maximum grip strength decreased significantly each year ($\upbeta$: − 0.03, 95% CI − 0.03, − 0.02 (SMI); $\upbeta$: − 0.21, 95% CI − 0.26, − 0.16 (maximum grip strength)) (Table 4). Neighborhood environmental factors that significantly affected the changes of SMI were not identified. With respect to grip strength, an average effect was found for bus stop density and distance to a community center, i.e., it was significantly lower in areas with a higher density of bus stops ($\upbeta$: − 0.61, 95% CI − 1.03, − 0.18) and significantly higher in areas far away from a community center ($\upbeta$: 0.53, 95% CI 0.11, 0.96). The changes in mean grip strength over time was only associated with residential density ($\upbeta$: 0.13, 95% CI 0.02, 0.24), which indicated that living in areas with high residential density would alleviate a decline in grip strength.

**Table 4:** Female—linear mixed model for SMI and grip strength by environment variable

	SMI		Grip strength	
	Model 1^a^	Model 2^b^	Model 1^a^	Model 2^b^
Variables	β (95% CI)	β (95% CI)	β (95% CI)	β (95% CI)
Year	− 0.03^*^ (− 0.03, − 0.02)	−	− 0.21^*^ (− 0.26, − 0.16)	− 0.1 (− 0.18, − 0.02)
Land slope (ref = Low)		− 0.01 (− 0.09, 0.07)		0.09 (− 0.33, 0.51)
Year*Land slope (ref = Low)		0 (− 0.02, 0.02)		− 0.05 (− 0.16, 0.06)
Bus stop density (ref = Low)		− 0.06 (− 0.14, 0.02)		− 0.61^*^ (− 1.03, − 0.18)
Year*Bus stop density (ref = Low)		0.01 (− 0.01, 0.04)		0.07 (− 0.04, 0.18)
Intersection density (ref = Low)		− 0.03 (− 0.11, 0.05)		− 0.3 (− 0.72, 0.12)
Year*Intersection density (ref = Low)		0.01 (− 0.02, 0.03)		0.01 (− 0.1, 0.12)
Residential density (ref = Low)		− 0.02 (− 0.1, 0.05)		− 0.26 (− 0.69, 0.16)
Year*Residential density (ref = Low)		0.01 (− 0.02, 0.03)		0.13^*^ (0.02, 0.24)
Distance to community center (ref = Close)		0.02 (− 0.06, 0.1)		0.53^*^ (0.11, 0.96)
Year*Distance to community center (ref = Close)		− 0.01 (− 0.03, 0.01)		− 0.05 (− 0.16, 0.06)

Environment factors were added separately into all models

^*^p < 0.05

^a^Model 1: time only model

^b^Model 2: conditional growth model by each environment factor adjusting for all covariates, i.e. age, town of residence, smoke, drink, physical activity, musculoskeletal disorders, cardiovascular disease, cerebrovascular diseases

### Number of new sarcopenia cases

Few new cases of sarcopenia (6 in males and 11 in females) were identified between 2016 and 2019 (Table 5).

**Table 5:** Number of incidence of sarcopenia between 2016 and 2019 by gender

	Male	Female
n	775	1140
Sarcopenia = No/Yes (%)	769/6 (99.2/0.8)	1129/11 (99.0/1.0)

## Discussion

The results of this study showed that older men living in areas far from community centers and women living in areas with high residential density had a less pronounced decline in SMI and grip strength over time compared to their counterparts. The effects of neighborhood environmental factors on both older men and women appeared small, yet this may have been due to the relatively short follow-up of 3 years; it is possible that a short follow-up only carries a subtle change of muscle mass and function in older adults over 60 years, which is supported by the low incidence of sarcopenia. It has been reported that decline in physical function and increased risk of sarcopenia in older adult populations requires a longer period to occur [35]. Additionally, subjects in this study had voluntarily opted to participate in group health examinations and they may therefore have been more conscious of their health and physically active. It was nonetheless shown that certain neighborhood environmental factors might affect longitudinal change of muscle mass and function for older men and women.

Previous studies have found that high social cohesion was associated with lower functional limitations [11, 14]. Living close to community centers can be hypothesized to increase the likelihood of engaging in social activities organized by community centers, and thus leading to increased social cohesion. However, our findings showed that living close to community centers had potentially detrimental effects on the decline in SMI among older men. Potential reasons for this are, firstly, living physically close to community centers does not necessarily mean participating in social activities or belonging to active groups. While it has been reported that proximity to resources was associated with social participation [36], whether or not individuals participated in these group activities should be assessed separately. Secondly, social norms within a tightly-knit group have been reported to be both health-promoting and health-damaging to older adults. Older men living physically close to community centers might be under more social pressure to get involved in activities, and that could lead to psychological stress and could negatively affect health because older men in general may be less willing to participate in social activities than older women [36]. Although further studies are needed to suggest policy implications, it may be possible to modify the types and/or frequency of activities held within community centers to be more beneficial to the residents. Thirdly, in our rural study areas, there are also smaller hubs besides community centers that organized social activities and it was not possible to capture these in our data. This might have biased our results in an unexpected direction.

High residential density has been found to contribute to the promotion of physical activity and improve certain health outcomes in older adults, which is consistent with our findings for older women [31, 32]. Residential density might be a more important health-promoting factor in women than in men, especially in rural settings. Moreover, it is less common for women to have a driving license than men; this is even more evident among older populations in rural parts of Japan. A previous study showed that more physical activity was observed among drivers than non-drivers as they were assumed to get to destinations where they can engage in exercise [37]. Another study showed that public transit access was more important to non-drivers than drivers among rural Japanese women [38]. However, the mechanisms of such associations were not examined in this study, and there is insufficient evidence to provide suggestions on policy. In addition, because it is difficult to change the residential density of an area, it may be more important to focus on other modifiable environmental factors, such as locations of commercial facilities. Commercial facilities, such as food stores and physical activity facilities (e.g. sports gym), are potentially modifiable via regulations in a relatively short time span compared to street or city layout, which require significant investment over long time periods. Several previous studies have examined these commercial facilities and found that they are associated with older adults’ nutritional status and physical activity level [39, 40].

This study has several limitations. First, the study subjects were not randomly sampled from each residential town. Our study subjects were limited to those who participated in the annual health examinations; thus, they might have been more conscious of their health than the general older adult population. This might explain why only subtle changes in SMI and grip strength as well as a low incidence of sarcopenia was observed in this study, in addition to a short follow-up period. Second, our study did not include aspects of individual and neighborhood factors relating to diet, an important determinant of muscle mass and strength in older adults [7]. Although the effect of diet on sarcopenia is not likely to be as firm as physical activity [24, 41], it should be investigated at the environmental level, such as locations of grocery and convenience stores, where intervention could possibly be implemented.

This study also has several strengths. First, this is the first study that has examined the potential effect of the neighborhood environment on muscle mass and function in more than 2000 older adults (≥ 60 years old) as well as in males and females separately, using a longitudinal study design. Most previous studies on neighborhood environments and physical function included middle age adults, such as 40 or 50 years old together with older adults and most studies were cross-sectional [10–15, 42–45]. Second, our study objectively measured both neighborhood environmental factors as well as muscle mass and function specific to sarcopenia. Although subjective measures are also important, they vary between individuals [46] and using objective measures is therefore more helpful to design a meaningful intervention. Third, our study targeted rural areas of Japan where the population aged more than 65 was already exceeding 35%. Despite the fact that rural populations are at a higher risk of chronic health conditions in general due to limited resources available [47, 48], studies targeting rural populations are limited [31, 32]. It is therefore crucial to study rural populations as a higher percentage of aging people are often found in rural settings, and this is likely to increase further in several countries across the world [49].

The Japanese government aims to establish community-based integrated care systems to enable elderly people to have an independent life in their own neighborhoods by 2025 [50]. Community centers play an important role for community-based services to elderly people and the findings of this study could thus be utilized when interventions are planned for rural elderly residents who often have difficulties in terms of access to many facilities and activities. Local governments may take measures to improve both the access and use of such facilities and activities based on objectively measured numerical indicators. In our study, we found that older men living far from community centers (≥ 1640 m) maintained their SMI better than those living close to community centers (< 1640 m). We also found that older women living in areas with more households (≥ 45) within a 1000 m network buffer maintained their grip strength better than those living in areas with fewer households (< 45) although an optimal threshold of residential density that can be beneficial for good health is unclear [51].

The findings of this study may suggest local governments, such as that in Shimane prefecture, to take a closer look on the specific types of facilities and activities offered at community centers and their potential effects on elderly people’s health rather than merely improving the proximity to community centers. Given the potential effects of the use of community centers for physical function improvements among older adults [33], it is, however, useful to know which areas and populations that need more focused interventions based on objectively collected data.

## Conclusions

Although the objectively measured neighborhood environmental factors in rural areas mostly had a quite limited effect on objectively measured muscle mass and muscle function decline in older adults there were certain neighborhood features that affected these outcomes more and some gender differences also appeared. This calls for further studies in other countries in order to investigate other modifiable environmental factors as well as following elderly individuals over a longer period.

## Supplementary information

**Additional file 1: Table S1.** Correlation between neighborhood environment factors among males. **Table S2.** Correlation between neighborhood environment factors among females.

## Data Availability

The data used in this paper include personal residential information as well as health related information. Therefore, the data are not publicly available due to privacy concerns. The data could be requested and available from the corresponding author on safe manner in accordance with the ethical policy statements of this study protocol.
