# Supplementary material for: Neighborhood environment and muscle mass and function among rural older adults: a 3-year longitudinal study
Source: Int J Health Geogr. 2020 Nov 25;19:51. doi: 10.1186/s12942-020-00247-9 (PMC7690178; doi:10.1186/s12942-020-00247-9)
Supplement: Supplementary file 1 — Additional file 1: Table S1. Correlation between neighborhood environment factors among males. Table S2. Correlation between neighborhood environment factors among females. [file 12942_2020_247_MOESM1_ESM.pdf]

Supplementary table 1. Correlation between neighbourhood environment factors among males.

	Land slope	Bus stop density	Intersection density	Residential density	Distance to community center
**Land slope**	1.00				
**Bus stop density**	-0.45	1.00			
**Intersection density**	-0.66	0.78	1.00		
**Residential density**	-0.49	0.71	0.72	1.00	
**Distance to community center**	0.33	-0.41	-0.44	-0.37	1.00

Supplementary table 2. Correlation between neighbourhood environment factors among females.

	Land slope	Bus stop density	Intersection density	Residential density	Distance to community center
**Land slope**	1.00				
**Bus stop density**	-0.43	1.00			
**Intersection density**	-0.64	0.76	1.00		
**Residential density**	-0.50	0.70	0.71	1.00	
**Distance to community center**	0.32	-0.42	-0.44	-0.37	1.00
